# 2-Eth­oxy-6-[(3-methyl­pyridin-2-yl)­imino­meth­yl]phenol

**DOI:** 10.1107/S1600536811012116

**Published:** 2011-04-07

**Authors:** Xiao-Ling Yuan

**Affiliations:** a College of Chemistry and Biology Engineering, Yichun University, Yichun 336000, People’s Republic of China

## Abstract

The title Schiff base compound, C_15_H_16_N_2_O_2_, was prepared by the condensation reaction of equimolar quanti­ties of 3-eth­oxy­salicyl­aldehyde with 2-amino-3-methyl­pyridine in methanol. The dihedral angle between the benzene ring and the pyridine ring is 2.6 (2)° and an intra­molecular O—H⋯N hydrogen bond generates an *S*(6) ring.

## Related literature

For background to Schiff bases, see: Sinha *et al.* (2008 ▶); Sonmez *et al.* (2010 ▶); Mohamed *et al.* (2010 ▶). For related structures, see: Wang & Shi (2008 ▶); Zhao *et al.* (2010 ▶); Karadağ *et al.* (2011) ▶; Bingöl Alpaslan *et al.* (2010 ▶).
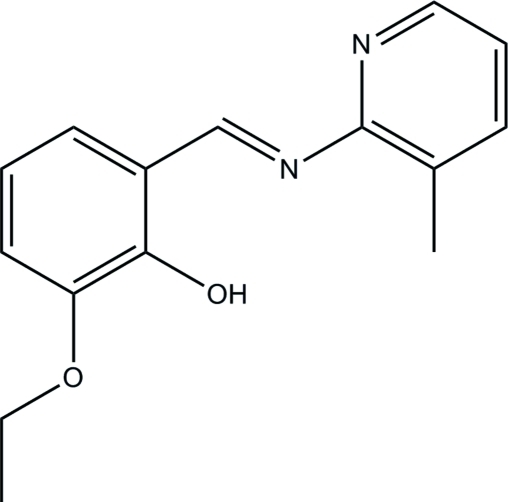


## Experimental

### 

#### Crystal data

C_15_H_16_N_2_O_2_
*M* *_r_* = 256.30
Monoclinic, 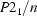

*a* = 4.820 (1) Å
*b* = 38.385 (3) Å
*c* = 7.207 (2) Å
β = 96.381 (2)°
*V* = 1325.1 (5) Å^3^
*Z* = 4
Mo *K*α radiation
μ = 0.09 mm^−1^
*T* = 298 K
0.17 × 0.15 × 0.15 mm

#### Data collection

Bruker SMART CCD area-detector diffractometer
Absorption correction: multi-scan (*SADABS*; Sheldrick, 1996 ▶) *T*_min_ = 0.985, *T*_max_ = 0.987
7773 measured reflections
2849 independent reflections
1265 reflections with *I* > 2σ(*I*)
*R*_int_ = 0.061

#### Refinement

*R*[*F* ^2^ > 2σ(*F* ^2^)] = 0.074
*wR*(*F* ^2^) = 0.200
*S* = 1.03
2849 reflections
175 parameters
H-atom parameters constrained
Δρ_max_ = 0.23 e Å^−3^
Δρ_min_ = −0.18 e Å^−3^

### 

Data collection: *SMART* (Bruker, 1998 ▶); cell refinement: *SAINT* (Bruker, 1998 ▶); data reduction: *SAINT*; program(s) used to solve structure: *SHELXS97* (Sheldrick, 2008 ▶); program(s) used to refine structure: *SHELXL97* (Sheldrick, 2008 ▶); molecular graphics: *SHELXTL* (Sheldrick, 2008 ▶); software used to prepare material for publication: *SHELXTL*.

## Supplementary Material

Crystal structure: contains datablocks global, I. DOI: 10.1107/S1600536811012116/hb5833sup1.cif

Structure factors: contains datablocks I. DOI: 10.1107/S1600536811012116/hb5833Isup2.hkl

Additional supplementary materials:  crystallographic information; 3D view; checkCIF report

## Figures and Tables

**Table 1:** Hydrogen-bond geometry (Å, °)

*D*—H⋯*A*	*D*—H	H⋯*A*	*D*⋯*A*	*D*—H⋯*A*
O1—H1⋯N1	0.82	1.87	2.590 (3)	146

**Fig. 1.**
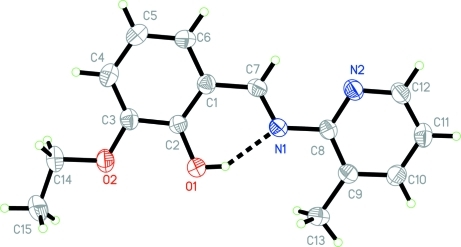
Molecular structure of the title compound with 30% probability ellipsoids.

## supplementary crystallographic information

### Comment

Much effort has been paid on the preparation, structures, and applications of Schiff bases (Sinha *et al.*, 2008; Sonmez *et al.*, 2010; Mohamed *et al.*, 2010). As a continuation of the work on the crystal structures of Schiff bases, the title new Schiff base compound, Fig. 1, is reported.

The whole molecule of the compound is approximately planar, with a mean deviation from the least squares plane through all 19 non-hydrogen atoms of 0.036 (2) Å; the dihedral angle between the C1–C6 benzene ring and the C8–C12/N2 pyridine ring is 2.6 (2)°. There is an intramolecular O1—H1···N1 hydrogen bond (Table 1), which helps the formation of the planarity of the molecule. The bond lengths and angles are comparable to those found in the similar Schiff base compounds (Wang & Shi, 2008; Zhao *et al.*, 2010; Karadağ *et al.*, 2011; Bingöl Alpaslan *et al.*, 2010).

### Experimental

Reagents and solvents used were of commercially available quality. A methanol solution (10 ml) of 2-amino-3-methylpyridine (0.1 mmol, 10.8 mg) was added to a stirred methanol solution (10 ml) of 3-ethoxysalicylaldehyde (0.1 mmol, 16.6 mg). After stirring for about 30 min at room temperature, the clear yellow solution was left to stand still in air. Yellow block-shaped crystals of the title compound were formed after slow evaporation of the solvent for a few days.

### Refinement

H atoms were placed in calculated positions and constrained to ride on their parent atoms, with C—H distances in the range 0.93–0.97 Å, O—H distance of 0.82 Å, and with *U*_iso_(H) set to 1.2*U*_eq_(C) and 1.5*U*_eq_(O1, C13 and C15).

### Crystal data

**Table:**

C_15_H_16_N_2_O_2_	*F*(000) = 544
*M**_r_* = 256.30	*D*_x_ = 1.285 Mg m^−^^3^
Monoclinic, *P*2_1_/*n*	Mo *K*α radiation, λ = 0.71073 Å
Hall symbol: -P 2yn	Cell parameters from 776 reflections
*a* = 4.820 (1) Å	θ = 2.5–24.5°
*b* = 38.385 (3) Å	µ = 0.09 mm^−^^1^
*c* = 7.207 (2) Å	*T* = 298 K
β = 96.381 (2)°	Block, yellow
*V* = 1325.1 (5) Å^3^	0.17 × 0.15 × 0.15 mm
*Z* = 4	

### Data collection

**Table:**

Bruker SMART CCD area-detector diffractometer	2849 independent reflections
Radiation source: fine-focus sealed tube	1265 reflections with *I* > 2σ(*I*)
graphite	*R*_int_ = 0.061
ω scan	θ_max_ = 27.0°, θ_min_ = 2.9°
Absorption correction: multi-scan (*SADABS*; Sheldrick, 1996)	*h* = −6→6
*T*_min_ = 0.985, *T*_max_ = 0.987	*k* = −49→49
7773 measured reflections	*l* = −9→9

### Refinement

**Table:**

Refinement on *F*^2^	Primary atom site location: structure-invariant direct methods
Least-squares matrix: full	Secondary atom site location: difference Fourier map
*R*[*F*^2^ > 2σ(*F*^2^)] = 0.074	Hydrogen site location: inferred from neighbouring sites
*wR*(*F*^2^) = 0.200	H-atom parameters constrained
*S* = 1.03	*w* = 1/[σ^2^(*F*_o_^2^) + (0.0714*P*)^2^ + 0.153*P*] where *P* = (*F*_o_^2^ + 2*F*_c_^2^)/3
2849 reflections	(Δ/σ)_max_ < 0.001
175 parameters	Δρ_max_ = 0.23 e Å^−^^3^
0 restraints	Δρ_min_ = −0.18 e Å^−^^3^

### Special details

**Table:**

Geometry. All e.s.d.'s (except the e.s.d. in the dihedral angle between two l.s. planes) are estimated using the full covariance matrix. The cell e.s.d.'s are taken into account individually in the estimation of e.s.d.'s in distances, angles and torsion angles; correlations between e.s.d.'s in cell parameters are only used when they are defined by crystal symmetry. An approximate (isotropic) treatment of cell e.s.d.'s is used for estimating e.s.d.'s involving l.s. planes.
Refinement. Refinement of *F*^2^ against ALL reflections. The weighted *R*-factor *wR* and goodness of fit *S* are based on *F*^2^, conventional *R*-factors *R* are based on *F*, with *F* set to zero for negative *F*^2^. The threshold expression of *F*^2^ > σ(*F*^2^) is used only for calculating *R*-factors(gt) *etc*. and is not relevant to the choice of reflections for refinement. *R*-factors based on *F*^2^ are statistically about twice as large as those based on *F*, and *R*- factors based on ALL data will be even larger.

### Fractional atomic coordinates and isotropic or equivalent isotropic displacement parameters (Å^2^)

**Table:**

	*x*	*y*	*z*	*U*_iso_*/*U*_eq_	
N1	0.6449 (5)	0.84753 (6)	0.0653 (4)	0.0495 (7)	
N2	0.9399 (6)	0.80518 (7)	0.2233 (4)	0.0604 (8)	
O1	0.3096 (5)	0.89291 (6)	−0.1048 (3)	0.0609 (7)	
H1	0.4207	0.8767	−0.0957	0.091*	
O2	−0.0665 (5)	0.94125 (6)	−0.0911 (3)	0.0691 (7)	
C1	0.3479 (6)	0.88351 (8)	0.2257 (5)	0.0508 (8)	
C2	0.2310 (6)	0.90021 (8)	0.0638 (4)	0.0458 (8)	
C3	0.0320 (7)	0.92615 (8)	0.0768 (5)	0.0550 (9)	
C4	−0.0520 (7)	0.93435 (9)	0.2466 (5)	0.0656 (10)	
H4	−0.1876	0.9514	0.2543	0.079*	
C5	0.0632 (8)	0.91749 (10)	0.4080 (5)	0.0728 (11)	
H5	0.0057	0.9234	0.5229	0.087*	
C6	0.2592 (7)	0.89243 (9)	0.3978 (5)	0.0625 (10)	
H6	0.3351	0.8811	0.5058	0.075*	
C7	0.5550 (7)	0.85676 (8)	0.2183 (5)	0.0530 (9)	
H7	0.6260	0.8458	0.3286	0.064*	
C8	0.8476 (6)	0.82108 (8)	0.0640 (5)	0.0482 (8)	
C9	0.9424 (7)	0.81326 (8)	−0.1071 (5)	0.0504 (8)	
C10	1.1400 (7)	0.78743 (9)	−0.1066 (5)	0.0603 (10)	
H10	1.2096	0.7813	−0.2174	0.072*	
C11	1.2348 (7)	0.77076 (9)	0.0558 (6)	0.0666 (10)	
H11	1.3680	0.7532	0.0568	0.080*	
C12	1.1299 (7)	0.78039 (9)	0.2162 (5)	0.0653 (10)	
H12	1.1950	0.7690	0.3263	0.078*	
C13	0.8314 (7)	0.83182 (9)	−0.2831 (4)	0.0679 (10)	
H13A	0.9225	0.8231	−0.3855	0.102*	
H13B	0.8669	0.8563	−0.2689	0.102*	
H13C	0.6340	0.8279	−0.3074	0.102*	
C14	−0.2849 (7)	0.96607 (9)	−0.0914 (5)	0.0684 (11)	
H14A	−0.2220	0.9861	−0.0165	0.082*	
H14B	−0.4439	0.9559	−0.0399	0.082*	
C15	−0.3642 (9)	0.97670 (11)	−0.2899 (6)	0.0961 (14)	
H15A	−0.2014	0.9847	−0.3428	0.144*	
H15B	−0.4995	0.9951	−0.2943	0.144*	
H15C	−0.4427	0.9571	−0.3600	0.144*	

### Atomic displacement parameters (Å^2^)

**Table:**

	*U*^11^	*U*^22^	*U*^33^	*U*^12^	*U*^13^	*U*^23^
N1	0.0489 (16)	0.0531 (16)	0.0459 (17)	0.0050 (13)	0.0022 (13)	0.0040 (13)
N2	0.0553 (18)	0.0665 (19)	0.0593 (19)	0.0105 (15)	0.0065 (15)	0.0123 (15)
O1	0.0654 (17)	0.0663 (16)	0.0507 (14)	0.0180 (12)	0.0054 (12)	0.0023 (12)
O2	0.0625 (15)	0.0719 (16)	0.0740 (17)	0.0234 (13)	0.0129 (13)	0.0121 (13)
C1	0.0462 (19)	0.051 (2)	0.054 (2)	0.0012 (16)	0.0037 (16)	−0.0032 (17)
C2	0.0430 (19)	0.0484 (19)	0.046 (2)	−0.0020 (15)	0.0049 (16)	−0.0013 (15)
C3	0.047 (2)	0.053 (2)	0.065 (2)	0.0026 (16)	0.0078 (18)	0.0068 (18)
C4	0.058 (2)	0.064 (2)	0.077 (3)	0.0098 (18)	0.018 (2)	−0.004 (2)
C5	0.074 (3)	0.084 (3)	0.064 (3)	0.013 (2)	0.022 (2)	−0.006 (2)
C6	0.065 (2)	0.077 (2)	0.045 (2)	0.008 (2)	0.0060 (18)	0.0018 (19)
C7	0.056 (2)	0.058 (2)	0.0441 (19)	0.0016 (17)	−0.0005 (17)	0.0029 (16)
C8	0.0435 (19)	0.0469 (19)	0.053 (2)	0.0002 (15)	0.0015 (16)	0.0034 (16)
C9	0.0461 (19)	0.052 (2)	0.052 (2)	−0.0050 (16)	0.0003 (16)	0.0011 (16)
C10	0.059 (2)	0.061 (2)	0.061 (2)	0.0038 (18)	0.0065 (19)	−0.0075 (18)
C11	0.059 (2)	0.059 (2)	0.082 (3)	0.0140 (18)	0.012 (2)	0.004 (2)
C12	0.065 (2)	0.069 (2)	0.063 (2)	0.016 (2)	0.0108 (19)	0.021 (2)
C13	0.073 (3)	0.081 (3)	0.049 (2)	0.007 (2)	0.0033 (19)	0.0014 (19)
C14	0.051 (2)	0.060 (2)	0.094 (3)	0.0129 (18)	0.007 (2)	0.006 (2)
C15	0.099 (3)	0.091 (3)	0.094 (3)	0.045 (3)	−0.007 (3)	0.008 (3)

### Geometric parameters (Å, °)

**Table:**

N1—C7	1.279 (4)	C7—H7	0.9300
N1—C8	1.410 (4)	C8—C9	1.394 (4)
N2—C12	1.326 (4)	C9—C10	1.375 (4)
N2—C8	1.332 (4)	C9—C13	1.500 (4)
O1—C2	1.342 (3)	C10—C11	1.367 (4)
O1—H1	0.8200	C10—H10	0.9300
O2—C3	1.378 (4)	C11—C12	1.363 (5)
O2—C14	1.420 (4)	C11—H11	0.9300
C1—C2	1.394 (4)	C12—H12	0.9300
C1—C6	1.399 (4)	C13—H13A	0.9600
C1—C7	1.437 (4)	C13—H13B	0.9600
C2—C3	1.393 (4)	C13—H13C	0.9600
C3—C4	1.368 (5)	C14—C15	1.496 (5)
C4—C5	1.392 (5)	C14—H14A	0.9700
C4—H4	0.9300	C14—H14B	0.9700
C5—C6	1.356 (4)	C15—H15A	0.9600
C5—H5	0.9300	C15—H15B	0.9600
C6—H6	0.9300	C15—H15C	0.9600
			
C7—N1—C8	120.5 (3)	C10—C9—C13	121.7 (3)
C12—N2—C8	117.5 (3)	C8—C9—C13	121.6 (3)
C2—O1—H1	109.5	C11—C10—C9	120.3 (3)
C3—O2—C14	117.8 (3)	C11—C10—H10	119.8
C2—C1—C6	119.6 (3)	C9—C10—H10	119.8
C2—C1—C7	121.0 (3)	C12—C11—C10	118.6 (3)
C6—C1—C7	119.3 (3)	C12—C11—H11	120.7
O1—C2—C3	118.4 (3)	C10—C11—H11	120.7
O1—C2—C1	122.2 (3)	N2—C12—C11	123.4 (3)
C3—C2—C1	119.3 (3)	N2—C12—H12	118.3
C4—C3—O2	125.6 (3)	C11—C12—H12	118.3
C4—C3—C2	120.0 (3)	C9—C13—H13A	109.5
O2—C3—C2	114.5 (3)	C9—C13—H13B	109.5
C3—C4—C5	120.7 (3)	H13A—C13—H13B	109.5
C3—C4—H4	119.7	C9—C13—H13C	109.5
C5—C4—H4	119.7	H13A—C13—H13C	109.5
C6—C5—C4	120.0 (3)	H13B—C13—H13C	109.5
C6—C5—H5	120.0	O2—C14—C15	107.2 (3)
C4—C5—H5	120.0	O2—C14—H14A	110.3
C5—C6—C1	120.4 (3)	C15—C14—H14A	110.3
C5—C6—H6	119.8	O2—C14—H14B	110.3
C1—C6—H6	119.8	C15—C14—H14B	110.3
N1—C7—C1	122.2 (3)	H14A—C14—H14B	108.5
N1—C7—H7	118.9	C14—C15—H15A	109.5
C1—C7—H7	118.9	C14—C15—H15B	109.5
N2—C8—C9	123.5 (3)	H15A—C15—H15B	109.5
N2—C8—N1	119.3 (3)	C14—C15—H15C	109.5
C9—C8—N1	117.2 (3)	H15A—C15—H15C	109.5
C10—C9—C8	116.6 (3)	H15B—C15—H15C	109.5

### Hydrogen-bond geometry (Å, °)

**Table:**

*D*—H···*A*	*D*—H	H···*A*	*D*···*A*	*D*—H···*A*
O1—H1···N1	0.82	1.87	2.590 (3)	146
